# Advancing human rights in patient care of Roma: access to health insurance in Macedonia

**DOI:** 10.1186/s40985-017-0064-5

**Published:** 2017-07-27

**Authors:** Nesime Salioska, Theodore T. Lee, Ryan Quinn

**Affiliations:** 1 ROMA S.O.S, Prilep, Republic of Macedonia; 2 Solomon Center for Health Law and Policy, Yale Law School, New Haven, CT USA; 3 Public Health Program, Open Society Foundations, New York, NY USA

**Keywords:** Health insurance, Access to health care, Macedonia, Right to health, Human rights in patient are, Human rights, State accountability, Roma

## Abstract

Roma in Macedonia suffer from dire health consequences due to economic factors, such as high rates of unemployment and poverty, and social factors, including discrimination by medical providers. Although Macedonia administers a public health care system for its citizens, Roma frequently lack access to this system in contravention of the rights to health and equality enshrined in Macedonia’s Constitution and international law. Applying a human rights in patient care (HRPC) framework to this problem, we discuss a facially neutral law that predicated access to health insurance for low-income citizens on the submission of a statement of income. This requirement created additional barriers to care, which we describe in this article. Even after the Constitutional Court declared the requirement invalid, the government failed to implement appropriate changes to the law in a timely manner. We argue this failure threatened the rule of law in the country and further marginalized and discriminated against Roma in violation of their human rights.

## Background

### The health of Roma in Macedonia

Roma are the largest ethnic minority in Europe and suffer marginalization in many spheres of life. Roma in Macedonia experience significantly higher rates of unemployment and poverty relative to the general population [1]. These factors translate into dire health consequences and are compounded by poor housing conditions in Roma communities and their geographic distance from health care facilities [2].

Official statistics about Roma in Macedonia are difficult to obtain since most government data do not record ethnicity. Moreover, the government has not conducted a census since 2002 [3]. The information that is available suggests that the health status of the Roma population is poor relative to the general population. In 2008 and 2009, the Institute of Public Health examined children in cities across the country and found that Roma children were below average in height, weight, and body mass index relative to their ages [4]. This study suggests that nutrition among Roma children is poor.

Other studies show that Roma face increased health risks and experience poor outcomes. Roma across Europe, including in Macedonia, are more likely to report unmet health needs than the general population, even after adjusting for demographic factors and socioeconomic factors [5]. A systematic review of the evidence found that Roma have higher mortality risks and Roma children have a greater prevalence of health risk factors such as low birth weight and lower vaccination coverage [6]. Studies in other countries suggest that life expectancy among Roma is lower than average [7]. In neighboring Serbia, infant mortality rates in Roma settlements are nearly twice as high as the national average [8].

The Macedonian government provides compulsory state-funded health care, managed by the Ministry of Health. However, as in neighboring Central and Eastern European countries, even when Roma in Macedonia are able to travel to health care facilities, they are routinely denied medical treatment or receive substandard care [9]. Discrimination by health providers is common. Roma patients in Macedonia, including pregnant women, have reported physical violence by health care professionals [10]. Remarks made by health providers about patients’ ethnicity suggest that this inferior treatment is due to racial prejudice [11], and some health professionals have negative underlying beliefs about Roma women [12]. Patients also report being required to pay for services that should have been free and if unable to pay being detained or having their documents confiscated [10].

One basis on which Roma in Macedonia are frequently denied health care is their lack of personal identification documents. A 2009 study focused on Roma in the Shuto Orizari region found that more than 30% of respondents had a period without health insurance and 14% had been denied health services as a consequence of not having appropriate documentation [13]. A 2016 United Nations International Children’s Emergency Fund (UNICEF) study reported that the most common reason Roma in Macedonia lack health insurance is a lack of identity documents (around 45% of those without health insurance) [14]. While the country’s Ministry of Labor and Social Policy has formed a task force to create a database of undocumented persons, no portion of the government budget has been allocated to this initiative and the Macedonian authorities rely instead on Roma-focused non-governmental organizations (NGOs) with external funding for information on individuals in need of identity documents [15].

This paper brings a human rights in patient care (HRPC) framework to bear on the issue of access to health insurance for Roma in Macedonia. The remainder of this section situates this issue within the academic literature on Roma health and human rights. The “Main text” section begins by situating access to health insurance within the HRPC framework. It then describes a specific barrier to health insurance for Roma and other impoverished communities in Macedonia, namely a facially neutral law requiring the annual submission of a signed statement of income in order to renew health insurance coverage. The “Main text” section concludes by analyzing the actions of the Macedonian authorities within the relevant human rights and legal frameworks.

### Methodology

The methodology underlying this paper was primarily qualitative in nature. The first author provided her findings based on having led the NGO campaign described in the “Main text” section, while the second and third authors integrated an HRPC analysis and cast the issues within the broader legal and human rights context.

### Review of the literature

It is important to situate the issue of access to health insurance for Roma in Macedonia within the broader academic literature on similar barriers to the realization of Roma health and human rights. These include barriers stemming from Macedonia’s citizenship regime and the conditionality tied to its visa liberalization and candidacy for EU membership. This literature highlights the need to address the health and human rights issues faced by Roma with the requisite complexity. Rather than being the “ultimate other” toward whom repressive policies are directly aimed [16], Roma often suffer unintended adverse effects from direct efforts to improve their condition and policies that appear neutral or universally progressive on their face.

The minority rights framework has so far failed to address the structural discrimination Roma face. Pogány argues that post-1990 minority rights regimes in Europe are simply not relevant for many Roma [17]. These regimes are largely unenforceable, and their focus on cultural, linguistic, and religious rights does not account for diversity within Roma communities. They also tend to overlook that the main problems affecting Roma concern their living conditions, access to public services, and education levels, as well as anti-Roma prejudice and hostility. On Pogány’s view, minority rights regimes have actually worsened Roma marginalization by casting them as a homogeneous minority and failing to address their structural socioeconomic challenges [17].

Efforts intended to benefit other minorities or society more broadly have also unintentionally undermined Roma rights. The first *Citizenship Law* following Macedonia’s declaration of independence in 1991 left many Roma and Albanians in the country without citizenship, not least because they could not prove continuous residency for the preceding 15 years [18]. As with the health insurance issues canvassed in this paper, this was largely a matter of lacking official documentation. Even after a series of amendments relaxed key requirements and brought Macedonia’s citizenship regime in line with European standards, Spaskovska reports persistent problems in the civil registration of Roma [18]. Sardelic deepens this analysis by casting Roma in Macedonia as the “collateral damage” of citizenship laws that targeted, if anyone, the more sizeable Albanian minority [16]. The result is that Roma in Macedonia evince a certain “forced in-betweenness” in a number of ways: they suffer the unintended and indirect impacts of tensions between the ethnic Macedonian majority and the Albanian minority and they are often considered “non-citizens” albeit without qualifying as “stateless” for the purposes of international protections [16].

Even attempts to address Roma rights can lead to backlash that ultimately undermines this aim. Kacarska describes how the European Commission’s efforts to incentivize Macedonia to improve the status of Roma rights—including by assuring their freedom of movement and their access to official documents—has played an important role in Macedonia’s visa liberalization and EU accession processes [19, 20]. Paradoxically, this has had two consequences inimical to Roma health and human rights. First, there has been a rise in xenophobic backlash by the ethnic Macedonian majority, who perceive special attention by European bodies to Roma rights as a sign of preferential treatment. Second, a rise in asylum seekers following visa liberalization—especially among Roma and Albanian nationals, in the case of Macedonia—has prompted EU member states and the European Commission to request that Macedonia and its neighbors restrict these groups’ freedom of movement. This has taken the form of profiling and harassment by Macedonian border police of Roma and other suspected “false asylum seekers” [19, 20]. This example illustrates that even well-intentioned efforts to improve Roma health and human rights sometimes have negative effects.

It is by building on this context that this paper considers a facially neutral law in Macedonia, which predicated access to health insurance on the annual submission of a signed statement of income. As Colombini et al. note, social health insurance schemes have sometimes worsened access for Roma and other impoverished groups, including in Macedonia, largely due to their lack of personal documentation [21]. This paper provides a specific instance of this phenomenon. This paper also builds on the existing literature by showing the adverse effects on Roma of requiring such documentation and analyzing the Macedonian government’s actions in light of NGO advocacy and the HRPC framework.

## Main text

### Health insurance coverage and the HRPC framework

The HRPC framework sheds light on the human rights dimensions of access to health insurance coverage. HRPC “widens out from the individual patient-provider relationship to examine systemic factors and state responsibility in the provision of patient care” [22]. The HRPC framework further calls for a focus on vulnerable populations in the formulation of health law and policy and “reveals issues of discrimination and social exclusion that often underlie abuse against patients” [22]. Additionally, its systemic approach attends to the rights and obligations of health care providers, recognizing that their duties can result in conflicts of “dual loyalty,” understood as conflicts related to their “simultaneous obligations, express or implied, to a patient and to a third party, often the state” [23]. Indeed, among the six common types of human rights violations that can result from dual loyalty conflicts, as identified by the International Dual Loyalty Working Group, is that of “[l] imiting or denying medical treatment or information related to treatment of an individual to effectuate the policy or practice of the state or other third party” [23].

Key human rights relevant to access to health insurance for Roma include the rights to the highest attainable standard of health and to non-discrimination and equality. These rights are protected by both Macedonia’s Constitution and international law.

The Macedonian Constitution includes expansive guarantees to health care and non-discrimination. With regard to health care, Article 39 of the Macedonian Constitution states that “[e]very citizen is guaranteed the right to health care” and that “citizens have the right and duty to protect and promote their own health and that of others” [24]. Article 34 further provides that “[c]itizens have the right to social security and social insurance, determined by law and collective agreement” [24]. The Constitution also guarantees equality through provisions such as Article 9, which states that citizens “are equal in their freedoms and rights, regardless of sex, race, colour of skin, national and social origin, political and religious beliefs, property and social status” [24].

International law also guarantees the rights to health and equality. Article 12 of the International Covenant on Economic, Social and Cultural Rights (ICESCR) “recognize[s] the right of everyone to the enjoyment of the highest attainable standard of physical and mental health” [25]. According to Article 2 of the ICESCR, these rights must be exercised without discrimination on the basis of “race, colour, sex, language, religion, political or other opinion, national or social origin, property, birth or other status” [25]. Additional treaties guarantee equality (Articles 2 and 26 of the International Covenant on Civil and Political Rights) [26], including in the area of health (Article 5 of the International Convention on the Elimination of All Forms of Racial Discrimination) [27]. Treaties also protect access to health care for specific populations like children (Article 24 of the Convention on the Rights of the Child) [28] and women (Article 12 of the Convention on the Elimination of All Forms of Discrimination against Women) [29].

The U.N. Committee on Economic, Social and Cultural Rights (CESCR) has elaborated on the ICESCR’s right to health, explaining that it must include accessibility. Accessibility includes the right to access health care without discrimination, “especially [for] the most vulnerable and marginalized sections of the population” [30]. Since adequate and affordable health care is critical to accessibility, “[s]ocial policies [that] disproportionately exclude patients from certain communities from access to health insurance” violate the right to health [22].

Under international human rights law, the Macedonian government should remove administrative obstacles tending to lead to unequal access to health care. The initial report adopted by the CESCR in 2006 expressed concern that many Roma did not have the personal documents required to access benefits such as social insurance and health care [31]. The CESCR recommended that the Macedonian government “take immediate steps, e.g. by removing administrative obstacles, to issue all Roma applicants with personal documents, with a view to ensuring their equal access to social insurance, health care and other benefits” [31]. The Committee on the Elimination of Racial Discrimination issued a report in 2007 that contained a similar recommendation [32]. These problems persist, however, and the CESCR again recommended in 2016 that the Macedonian government “take all measures necessary to issue identity cards to all Roma” [33]. The CESCR also noted that insufficient funding for the health sector limited access to health services for Roma and people in rural areas [33].

Equality principles also apply in the patient-care setting. The Committee on the Rights of the Child (CRC) wrote in a 2014 report that despite near-universal health insurance coverage in Hungary, “a number of persons who belong to the Roma community continue to be denied health services, including emergency aid services, and are discriminated against by health practitioners” [34]. This CRC report applied equality principles to the provision of health services including the importance of non-discrimination by providers. The Committee recommended the government “provide health-care services to all children within its territory without any discrimination” [34]. Although the subject of this article is Roma in Macedonia (and not Hungary), the CRC report emphasizes the obligations governments across Europe have with respect to the health of Roma.

### The termination of health insurance coverage among Roma in Macedonia

In April 2013, the Prilep-based NGO ROMA S.O.S. was consulted by a woman who had given birth at the P.H.I. General Hospital Borka Taleski and, upon being discharged, had been billed 13,000 denars—the uninsured cost of the medical services she had received. This invoice was perplexing because the patient had received regular confirmation of her coverage under Macedonia’s Health Insurance Fund (HIF) up to the preceding month. Upon investigation, ROMA S.O.S. discovered that her coverage had been terminated because she had not filed a signed statement of income for the previous year. This client was the first of 280 similar cases of terminated health insurance coverage documented by ROMA S.O.S. in April and May 2013. Upon submitting an access-to-information request to the HIF, ROMA S.O.S. learned that only 52.9% (120,255) of those individuals eligible for exemption from payment for health insurance had submitted a statement of income and thus been able to maintain their coverage [35].

Two years prior, amendments to Macedonia’s *Law on Contributions to Compulsory Social Insurance* had introduced a requirement that low-income and unemployed citizens submit signed statements of income each year to “re-register” for continued coverage [36]. However, the Constitutional Court of Macedonia abolished this requirement in November 2012 finding that the HIF itself was responsible for verifying the incomes of insured persons through the country’s Public Revenue Office [37]. As ROMA S.O.S. and its clients discovered, this decision was far from fully implemented; the Macedonian government had amended the *Law* only to remove the obligation of the Ministry of Finance to prescribe the form and its contents [38].

As a result, in January 2013 the HIF once again called for all low-income and unemployed citizens to re-register for their health insurance coverage by submitting a signed statement of income showing that their 2012 income had not exceeded 96,600 denars. Many of these individuals were not notified, however, and were not aware of the HIF’s new requirement. As a result, many people lost health insurance coverage because they did not comply in a timely manner, raising another administrative obstacle to health insurance. In many cases, these individuals only learned of this requirement when they next attempted to access health care services. At that time, they were informed that their health insurance coverage had been suspended and that they needed to pay the full cost of the health care services and medications they required—as well as an additional administrative fee to obtain the documentation required to re-register [35].

In response, ROMA S.O.S. formed a coalition with six other NGOs from across Macedonia (Humanitarian and Charitable Association of Roma Delcevo, the Association for Roma Community Development, National Roma Centrum, Health Education and Research Association, Macedonian Anti-Poverty Platform, and Association for Emancipation, Solidarity and Equality of Women). The coalition made submissions to the Ministry of Finance, the Ministry of Health, the Health Committee, and the Ombudsman, an independent official responsible for protecting citizens’ rights, asking these bodies to examine the implementation of the Constitutional Court’s decision. Responding in September 2013, the ministries insisted that although they were aware of the great number of people who had lost their health insurance coverage for failure to submit a statement of income, it was not an onerous obligation to comply with this requirement. The Ombudsman, by contrast, agreed with ROMA S.O.S. and its NGO partners that the court’s decision had not been properly implemented and that immediate legislative amendments were required [35].

Over the following months, ROMA S.O.S. and its civil society partners met with political leaders and generated public awareness of the issue through press conferences, public discussions, and engagement with news media [35]. This advocacy resulted in the unanimous adoption by the Assembly of the Republic of Macedonia of an amendment to the *Law on Contributions to Compulsory Social Insurance* in December 2014*.* This amendment removed the requirement that low-income and unemployed individuals submit an annual statement of income in order to retain their health insurance coverage [39]. Because the HIF does not collect data by ethnicity, it is difficult to determine exactly how many Roma benefited from this intervention. However, an unofficial estimate by ROMA S.O.S. using conservative assumptions suggests that about 32,000 Roma benefited from the legal amendment. (This estimate uses the latest available census data, which reported a Roma population of nearly 54,000 [3]. This calculation also makes the assumptions that the primary beneficiaries were unemployed people and that approximately 60% of the Roma population is unemployed.)

Although the Macedonian government had lifted the statement of income requirement, in early 2015, news emerged that 6760 low-income and unemployed individuals who *had* filed statements of income in previous years had been criminally charged with providing a document with false information; data from the Public Revenue Office revealed that these individuals’ income was in fact higher than they had reported [35]. The reasons for this underreporting, according to personal communications with ROMA S.O.S. and its clients, included that these individuals could not understand the forms and were simply told to circle one of the available options, that they were personally obliged to calculate their income for the preceding year long before the Public Revenue Office itself had generated this data, and that it was unclear whether net income or gross income was to be reported.

There was a marked lack of uniformity in how public prosecutors pursued criminal charges. Charges against 1789 individuals were resolved in brief proceedings conducted in trial courts. In most of these cases, the accused were completely unaware that they had been charged; this was the case for several Roma whose residential addresses had recently changed. They were convicted in absentia and sentenced to 3 months in prison, 1 year of probation, and a fine of 3000 denars. In another 1524 cases, however, the charges were dismissed and no proceedings held on the basis that they were not criminal cases to be prosecuted ex officio, that the cases were obsolete or of minor importance, that the person had no income at all, or that the Public Revenue Office had wrongly imputed a family member’s income to the accused [35].

In tandem with these proceedings, the HIF began to request reimbursement from the accused for the health care services they had used in the years for which they had submitted “false” statements of income. The agency proceeded with these claims without formally filing civil lawsuits and despite not demonstrating pecuniary losses. These charges and claims were ultimately dropped by legislative amendment in June 2015 [40], as a result of advocacy by NGOs.

### A human rights analysis of the Macedonia case

This section provides a health and human rights analysis of the situation described above, examining both the regulatory framework and the actions and inaction of Macedonian authorities.

The country’s *Law on Health Insurance* assures mandatory coverage of primary health care services for citizens [41]. (Citizens can obtain coverage for other services if they have the means to pay for it.) The separate *Law on Contributions to Compulsory Social Insurance* regulates the levels of contributions required for mandatory insurance covering primary health care; it specifies that citizens are exempted from payment if they earn less than 96,600 denars per year and, as of 2011, required such individuals to submit a signed statement of income each year in order to maintain their coverage [36]. Contributions by citizens whose income surpasses this threshold make up most of the funding for health care in Macedonia. The HIF covers 80% of health care costs for insured citizens, who must pay the remaining 20% at the point of service.

The requirement to submit an annual statement of income may not seem to represent a significant burden; however, most Roma and other low-income or unemployed individuals are among those least likely to be made aware of such a requirement or to have ready access to the required documentation. Insofar as the state failed to account for the hurdles and already-dire health outcomes experienced by Roma when it introduced this requirement, it fell short of HRPC standards. Moreover, the statement of income requirement and its consequences for health insurance coverage indirectly place health care providers in a dual loyalty conflict, pitting their duty to abide by the law against their professional and ethical duties to provide medical treatment to all patients who visit their facilities. Macedonia’s constitutional guarantees of health care and social insurance were unduly tempered by the statement of income requirement because those segments of the population who were least likely to possess or file such documentation—including Roma—were denied their constitutional rights on the basis of that very vulnerability. The requirement also discriminated, in effect if not in intent, against Roma and other low-income and unemployed persons notwithstanding the anti-discrimination provisions of the *Law on Health Insurance*.

The right to health in the ICESCR imposes three obligations on governments: (1) to *respect* by refraining from interfering with enjoyment of the right to health; (2) to *protect* by taking steps to prevent third parties from threatening the right to health; and (3) to *fulfill* by adopting measures appropriate to fully realize the right to health [30]. The Macedonian government’s actions failed to meet these obligations because it interfered with the ability of Roma and low-income people to access health insurance, failed to protect these vulnerable groups from denial of service, and did not proactively adopt measures to promote the right to health among Roma. Although the law did not specifically target these groups for discrimination, laws that are facially neutral can have discriminatory effects that contravene international law. For example, the Special Rapporteur on Migrants found a facially neutral law in Japan that made national health insurance available to foreign nationals with a residence visa for 12 months or longer discriminated against migrants [42]. Similarly, the statement of income requirement imposed a neutral standard to receive access to health insurance; however, this standard’s significant burden on Roma and other low-income citizens had discriminatory effects prohibited by international law.

In addition, the Macedonian authorities undermined the rule of law by failing to respect the decision of the Constitutional Court invalidating the statement of income requirement. Despite awareness of the requirement’s impact and persistent pressure by ROMA S.O.S. and its civil society partners, the HIF again issued a call in February 2014 for signed statements of income from all low-income and unemployed citizens. Once more, ROMA S.O.S. found that little more than one-half of eligible individuals (55.8%, or 135,583 people) had submitted the statement, and thus, health insurance coverage was terminated for all others (44.2%, or 107,502 people) [35].

The government’s attempts to hold people criminally liable for errors on their statements of income also abridged the right to health insurance. These spurious criminal charges and civil claims were brought against members of socially excluded groups who attempted to comply with a law that had discriminatory effects on them. By deepening the marginalization of low-income and unemployed persons in pursuing these claims, the Macedonian government further violated the rights to health and equality in Macedonia’s Constitution, the ICESCR, and other international treaties.

This series of events illustrates the role that advocacy can play in enforcing international norms and benefiting marginalized people. Formal enforcement of international human rights is difficult, and without the threat of punishment, governments often lack incentives to comply with their obligations. International bodies such as the CESCR have recognized the human rights issues plaguing Roma in Macedonia. Nevertheless, these bodies alone struggle to generate the coalition necessary to address structural discrimination and the effects of facially neutral laws such as the statement of income requirement or criminal liability for false statements. In the Macedonia case, intra-country pressure by NGOs filled the gap and played an important role in correcting the problem.

Ultimately, the Macedonian government is the actor that adopted the changes and will need to act in the future to ensure the human rights of Roma. The government should address the structural challenges and discrimination that continue to prevent Roma from accessing health insurance. Since the changes to the law in 2014, citizens who are unemployed or have low incomes, including Roma, have not faced additional administrative obstacles in realizing their right to health care. However, despite the amendments to the health insurance law, Roma remain unable to fully exercise their human rights. Recent studies confirm that lack of identity documents remains the most common obstacle to health insurance for Roma in Macedonia [14]. The CESCR has recognized the ongoing “structural discrimination against Roma,” including Macedonia’s lack of effective measures to address employment, housing, education, and nutrition needs [33]. The Macedonian government should take action to address these concerns.

## Conclusion

Roma in Macedonia face significant barriers to attaining the rights to health and equality guaranteed by Macedonia’s Constitution and international law. Applying a HRPC framework to this problem reveals the barriers to health insurance created by a facially neutral law requiring a statement of income. These barriers violated Macedonia’s Constitution and were suspect under international law. After the Constitutional Court declared the requirement invalid, the government failed to implement appropriate changes to the law in a timely manner. This failure threatened the rule of law and further marginalized and discriminated against Roma in violation of their human rights.

This episode confirmed that legislative tools can both create and remedy obstacles to accessing health care. Facially neutral laws such as the statement of income requirement can have disproportionate negative impacts on minority groups such as Roma. Given the obligations governments have under international human rights law, they should consider this possibility when designing and implementing social programs—even when those programs are intended to benefit society broadly. Policymakers and commentators must critically evaluate even seemingly progressive programs for their impact on marginalized groups.

Though this particular barrier to health insurance has been addressed, structural challenges and discrimination remain. Even assessing the extent of these problems is difficult because of the lack of official statistical information; the CESCR has recommended that the government improve its data collection to allow reliable and robust analysis of the situation of Roma in the country [33]. The Macedonian government should take action to address these obstacles to Roma fully realizing their economic, social, and cultural rights as well as the rights guaranteed under Macedonia’s Constitution.

## Abbreviations

CESCR: U.N. Committee on Economic, Social and Cultural Rights
CRC: Committee on the Rights of the Child
HRPC: Human rights in patient care
ICESCR: International Covenant on Economic, Social and Cultural Rights
NGO: Non-governmental organization
UNICEF: United Nations International Children’s Emergency Fund

## References

[1] Bojadjieva A. Roma Inclusion Index 2015 Budapest: Decade of Roma Inclusion Secretariat Foundation; 2015. http://www.rcc.int/romaintegration2020/files/user/docs/Roma%20Inclusion%20Index%202015.pdf.

[2] Pavlovski B, Antik D, Friscik J, Gelevska M, Misev S, Kasapinov B (2014). We are all human: health care for all people regardless of their ethnicity.

[3] Republic of Macedonia State Statistical Office (2002). Census of population, households and dwellings in the Republic of Macedonia, 2002 - Book XIII.

[4] Institute of Public Health of the Republic of Macedonia. [Извештај за реализација на програмата за превентивна здравствена заштита на Република Македонија во 2009 година.] Report on the implementation of the program for preventive health care of the Republic of Macedonia in 2009. Skopje: Institute of Public Health; 2009.

[5] Arora VS, Kühlbrandt C, McKee M (2016). An examination of unmet health needs as perceived by Roma in Central and Eastern Europe. Eur J Pub Health.

[6] Cook B, Wayne GF, Valentine A, Lessios A, Yeh E (2013). Revisiting the evidence on health and health care disparities among the Roma: a systematic review 2003–2012. Int J Public Health.

[7] Fundación Secretariado Gitano (2012). Health and the Roma community, analysis of the situation in Europe: Bulgaria, Czech Republic, Greece, Portugal, Romania, Slovakia, Spain.

[8] Statistical Office of the Republic of Serbia and UNICEF (2014). Serbia multiple indicator cluster survey and Serbia Roma settlements multiple indicator cluster survey, 2014.

[9] Ezer T, Cohen J, Quinn R (2014). The problem of torture in health care. Torture in healthcare settings: reflections on the Special Rapporteur on Torture’s 2013 thematic report.

[10] Abdikeeva A, Ezer T, Covaci A (2013). Assessing legal advocacy to advance Roma health in Macedonia, Romania, and Serbia. Eur J Health Law.

[11] European Roma Rights Centre (2006). Ambulance not on the way: the disgrace of health care for Roma in Europe.

[12] Watson HL, Downe S (2017). Discrimination against childbearing Romani women in maternity care in Europe: a mixed-methods systematic review. Reproductive Health.

[13] Pavlovski B (2009). Health, healthcare and the impact on the health of the Roma in the Republic of Macedonia.

[14] UNICEF (2016). Assessment of barriers to health insurance access for Roma families.

[15] Open Society Foundations (2017). Roma health rights in Macedonia, Romania, and Serbia: a follow-up assessment of legal advocacy efforts.

[16] Sardelic J (2015). Romanic minorities and uneven citizenship access in the post-Yugoslav space. Ethnopolitics.

[17] Pogány I (2006). Minority rights and the Roma of Central and Eastern Europe. Human Rights Law Review.

[18] Spaskovska L. Macedonia’s nationals, minorities and refugees in the post-communist labyrinths of citizenship. CITSEE Working Paper Series 2010/05.

[19] Kacarska S. Europeanisation through mobility: visa liberalisation and citizenship regimes in the Western Balkans. CITSEE Working Paper Series 2012/21.

[20] Kacarska S (2012). Minority policies and EU conditionality—the case of the Republic of Macedonia. Journal on Ethnopolitics and Minority Issues in Europe.

[21] Colombini M, Rechel B, Mayhew S (2012). Access of Roma to sexual and reproductive health services: qualitative findings from Albania, Bulgaria and Macedonia. Global Public Health.

[22] Cohen J, Ezer T (2013). Human rights in patient care: a theoretical and practical framework. Health Hum Rights.

[23] International Dual Loyalty Working Group (2002). Dual loyalty and human rights in health professional practice: proposed guidelines & institutional mechanisms.

[24] The Republic of Macedonia. Устав на Република Македониjа [Constitution of the Republic of Macedonia]. 2001.

[25] United Nations General Assembly (1966). International Covenant on Economic, Social and Cultural Rights.

[26] United Nations General Assembly (1966). International Covenant on Civil and Political Rights.

[27] United Nations General Assembly (1965). International Convention on the Elimination of All Forms of Racial Discrimination.

[28] United Nations General Assembly (1989). Convention on the Rights of the Child.

[29] United Nations General Assembly (1979). Convention on the Elimination of All Forms of Discrimination against Women.

[30] United Nations Committee on Economic (2000). Social and Cultural Rights.

[31] United Nations Committee on Economic (2008). Social and Cultural Rights.

[32] United Nations Committee on the Elimination of Racial Discrimination (2007). Concluding observations of the Committee on the Elimination of Racial Discrimination: the former Yugoslav Republic of Macedonia.

[33] United Nations Committee on Economic, Social and Cultural Rights (2016). Concluding observations on the combined second to fourth periodic reports of the former Yugoslav Republic of Macedonia.

[34] United Nations Committee on the Rights of the Child (2014). Concluding observations on the combined third, fourth and fifth periodic reports of Hungary.

[35] ROMA S.O.S (2016). Project “Roma Health—Basic Human Right”: law initiative 2013, 2014, 2015.

[36] Службен Весник на Република Македонија Број 53 [Official Gazette of the Republic of Macedonia no. 53], Закон за изменување и дополнување на Законот за придонеси од задолжително социјално осигурување [Law Amending the Law on Contributions to Compulsory Social Insurance] 25–27. 2011.

[37] Уставен Суд на Република Македонија [Constitutional Court of Republic of Macedonia] 157/2011–0-1. 2012.

[38] Службен Весник на Република Македонија Број 15 [Official Gazette of the Republic of Macedonia no. 15], Закон за изменување и дополнување на Законот за придонеси од задолжително социјално осигурување [Law Amending the Law on Contributions to Compulsory Social Insurance] 7–9. 2013.

[39] Службен Весник на Република Македонија Број 180 [Official Gazette of the Republic of Macedonia no. 180], Закон за изменување и дополнување на Законот за придонеси од задолжително социјално осигурување [Law Amending the Law on Contributions to Compulsory Social Insurance] 8–10. 2014.

[40] Службен Весник на Република Македонија Број 98 [Official Gazette of the Republic of Macedonia no. 98], Закон за дополнување на Законот за здравственото осигурување [Law Amending the Law on Health Insurance] 14–16. 2015.

[41] Во Законот за здравственото осигурување [Law on Health Insurance] (“Службен Весник на Република Македонија” број [“Official Gazette of the Republic of Macedonia” nos.] 25/2000, 34/2000, 96/2000, 50/2001, 11/2002, 31/2003, 84/2005, 37/2006, 18/2007, 36/2007, 82/2008, 98/2008, 6/2009, 67/2009, 50/2010, 156/2010, 53/2011, 26/2012, 16/2013, 91/2013, 187/13, 43/2014, 44/2014, 97/2014, 112/2014, 113/2014, 188/2014, 20/2015, 61/2015, 98/2015, 129/2015, 150/2015, 154/2015, 192/2015, 217/2015, 27/2016, 37/2016, 120/2016 и [and] 142/2016).

[42] Bustamente J (2011). Report of the Special Rapporteur on the human rights of migrants, on his mission to Japan (23–31 March 2010).

